# Prevalence and determinants of depressive symptoms in patients with Chagas cardiomyopathy and predominantly preserved cardiac function

**DOI:** 10.1590/0037-8682-0123-2020

**Published:** 2020-11-06

**Authors:** Whesley Tanor Silva, Matheus Ribeiro Ávila, Lucas Frois Fernandes de Oliveira, Pedro Henrique Scheidt Figueiredo, Vanessa Pereira Lima, Alessandra de Carvalho Bastone, Fábio Silva Martins da Costa, Mauro Felippe Felix Mediano, Henrique Silveira Costa, Manoel Otávio da Costa Rocha

**Affiliations:** 1 Universidade Federal dos Vales do Jequitinhonha e Mucuri, Faculdade de Ciências Biológicas e da Saúde, Departamento de Fisioterapia, Diamantina, MG, Brasil.; 2 Universidade Federal de Minas Gerais, Escola de Medicina, Curso de Pós-Graduação em Infectologia e Medicina Tropical, Belo Horizonte, MG, Brasil.; 3 Fundação Oswaldo Cruz, Instituto Nacional de Infectologia Evandro Chagas, Rio de Janeiro, RJ, Brasil.

**Keywords:** Chagas disease, Chagas cardiomyopathy, Depressive disorder, Exercise test, Activities of daily living, Mental health

## Abstract

**INTRODUCTION:**

Chagas cardiomyopathy (ChC) is highly stigmatized, and the presence of depressive symptoms may be a common feature. However, its determinants remain unclear. Therefore, the present study aimed to verify the prevalence of depression and the clinical, echocardiographic, functional, and quality of life factors associated with depressive symptoms in patients with ChC and predominantly preserved cardiac function.

**METHODS:**

Thirty-five patients with ChC (aged 40 to 60 years, 66% men, NYHA I-III) were evaluated by echocardiography, cardiopulmonary exercise testing, 6-minute walk test (6MWT), and Mini-Mental State Examination. Physical activity level was assessed using the Human Activity Profile (HAP) and health-related quality of life was assessed using the Short-Form Health Survey (SF-36). Depressive symptoms were evaluated using the Beck Depression Inventory. A cutoff point greater than 9 was indicative of depression.

**RESULTS:**

Depression was detected in 13 patients (37%). In the univariate analysis, female sex, NYHA functional class, body mass index, HAP score, mental summary of SF-36, peak oxygen uptake, and 6MWT distance were associated with depressive symptoms. The final model showed that only the HAP score (B = -0.533; 95% confidence interval [CI]: -0.804 to -0.262) and SF-36 mental summary (B = -0.269; 95% CI: -0.386 to -0.153) remained as independent predictors of depressive symptoms in patients with ChC.

**CONCLUSIONS:**

Depression was prevalent in patients with ChC and predominantly preserved cardiac function. Physical activity and mental health were independent risk factors for depressive symptoms.

## INTRODUCTION

Depression is a result of the complex interactions among social, psychological, and biological factors^1^. It is a very prevalent health condition that stands out for being the leading cause of “lost years” due to disability worldwide^1^ ^,^ ^2^. This becomes even more important when it is associated with another disease^1^, such as some cardiovascular disorders^3^ ^,^ ^4^, in which the presence of depressive symptoms may contribute to a worse prognosis related to morbidity, mortality, poor treatment adherence, and functional decline^5^. In the context of cardiovascular disease, Chagas cardiomyopathy (ChC) stands out because individuals with this disease often face social and economic discrimination due to the stigma of poverty and the fear of sudden cardiac death^6^.

Chagas disease is an infection caused by the protozoan *Trypanosoma cruzi*. The disease is endemic in Latin America, with an increased prevalence in non-endemic countries such as the United States and some regions of Europe due to migration to these areas^7^ ^,^ ^8^. Among patients with this disease, 30% to 40% experience the cardiac form of the infection, which is characterized by constant inflammation, loss of cardiac muscle fibers, systolic dysfunction, functional impairment, and worse prognosis than other cardiopathies^9^ ^,^ ^10^, which in turn may be associated with mental torment and the onset of depressive symptoms^3^ ^,^ ^11^.

In patients with Chagas disease, most studies and clinicians have focused on the body structure and function components such as cardiac function^12^ ^,^ ^13^, myocardial dilation^14^, and functional capacity^10^. Despite the importance of the presence of depressive symptoms in Chagas disease and the impact of these symptoms on clinical management, few studies have investigated these symptoms. Forsythy^15^ reported that patients with Chagas disease typically experience emotional stress since the diagnosis of the disease. Ozaki and de Almeida^16^ found that 41.63% of the patients with Chagas disease presented with depressive symptoms (n = 110; comprising indeterminate, cardiac, digestive, and mixed forms). Despite the high prevalence, the determinants of depressive symptoms remain unclear, especially in the early stages of heart disease. Early recognition of the determinants of depressive symptoms may aid in clinical management and improve the health-related quality of life of this neglected population. 

Therefore, this study aimed to verify the prevalence of depressive symptoms and their clinical, echocardiographic, and functional determinants in patients with ChC and predominantly preserved cardiac function.

## METHODS

This was a cross-sectional study conducted at the outpatient clinic for Chagas disease in an endemic area in the state of Minas Gerais, Brazil. Clinically stable patients with ChC were recruited between June 2019 and March 2020. The research was conducted in accordance with the principles of the Declaration of Helsinki^17^ and was approved by the Institutional Ethics Committee (protocol CAAE 16379719.5.0000.5108, 07/22/2019). All the patients were instructed about the objectives and methods of the study and provided written informed consent before participating.

A minimum of two different serological tests (indirect immunofluorescence, enzyme-linked immunosorbent assay, or indirect hemagglutination) for antibodies against *Trypanosoma cruzi* were required for the diagnosis of Chagas disease. Serological tests were performed simultaneously, and the diagnosis was confirmed when both methods were reactive. To be included in the present study, patients were required to be between 40 and 60 years of age and to have electrocardiographic and/or echocardiographic abnormalities compatible with ChC according to I Latin American Guideline^18^. Patients with ChC who presented with electrocardiographic or echocardiographic changes but with normal global ventricular function were classified as stage B1. Patients with ChC and global ventricular dysfunction but without previous or current signs and symptoms of chronic heart failure were classified as having stage B2. Exclusion criteria were the use of antidepressant medications, the presence of cardiopathy by other causes, cognitive impairment as determined by the Mini Mental State Examination, and inability to perform the procedures.

All patients underwent clinical examination, echocardiography, and cardiopulmonary exercise testing (CPET), followed by the 6-minute walking test (6MWT). The Mini-Mental State Examination, Beck Depression Inventory (BDI), Human Activity Profile (HAP), and Short-Form Health Survey (SF-36) questionnaires were administered. The researchers responsible for each evaluation were blinded to the results of the other tests.

### HAP

The HAP is a self-reported questionnaire composed of 94 activities that exhibit variable low- to high-energy requirements, in which the order of the activities is based on their estimated metabolic equivalents. Higher scores were associated with higher physical activity levels^20^. The HAP has been used to evaluate physical activity in research and clinical settings for a variety of health conditions, including ChC^19^ ^,^ ^20^. 

### BDI

The BDI, an inventory already used in patients with ChC^16^, was used to verify the presence of depressive symptoms. The inventory comprises 21 questions that address the main types of symptoms and attitudes associated with depression, namely, mood, pessimism sense of failure, self-dissatisfaction, guilt, sense of punishment, self-dislike, self-accusations, suicidal ideas, crying, irritability, social withdrawal, indecisiveness, distortion of body self-image, work difficulty, insomnia, fatigability, appetite, weight loss, somatic occupation, and loss of libido. The patient evaluated the intensity of the symptoms on a scale of 1 to 3, according to Gorenstein^21^. According to the BDI score, the patients were classified into the following levels of depression: no depression or minimal depression = 0-9, mild to moderate depression = 10-18, moderate to severe depression = 19-29, and severe depression = 30-63, as validated for the Brazilian population^22^.

### CPET

CPET is the gold standard in functional evaluation^23^ and in the present study, it was performed on a treadmill with the metabolic analysis system MetaLyzer 3B (Cortex Medical, Leipzig, Germany). The peak VO2 and minute ventilation-carbon dioxide production (VE/VCO2) slope were used as the primary endpoints in the functional evaluation and were obtained according to current guidelines^24^.

### Echocardiographic Evaluation

An echocardiogram was performed to verify the left ventricular ejection fraction (LVEF) and left ventricular end-diastolic diameter (LVDd), according to the American Society of Echocardiography^25^. The LVEF was obtained using the modified Simpson rule.

### Mini-Mental State Examination

The Mini-Mental State Examination was used to assess the patients' cognitive functions. It is a questionnaire composed of 20 questions related to domains such as attention, language, word memory, and orientation in time and space, and is used by clinicians and researchers to screen for cognitive impairment. The final score varied from 0 to 30, and a result of less than or equal to 23 was considered to indicate cognitive impairment^26^ ^,^ ^27^.

### SF-36 Questionnaire

SF-36 was used to assess health-related quality of life (HRQoL). This questionnaire consists of eight domains addressing physical (functional capacity, physical aspects, pain, vitality, and general health) and mental (social aspects, and emotional and mental health) aspects, and the final score varied from 0 (the most affected) to 100 (without commitment). The questionnaire has been previously translated to Brazilian Portuguese^28^. In the present study, the answers of the physical and mental components were summarized.

### 6MWT

The 6MWT is a field test widely used for the functional evaluation of patients with ChC^29^ ^-^ ^31^. The test was performed according to American Thoracic Society guidelines^32^, and the volunteers were instructed to walk at the highest possible speed without running for over a distance of 30 m in a period of 6 min. Standardized words of encouragement were provided every minute, and the test was applied twice with a 15-min rest interval. The target variable was the longest distance covered by the patient.

### Statistical Analysis

Statistical analyses were performed using Statistical Package for the Social Sciences (SPSS), version 17.0. Normal distribution of the data was verified using the Kolmogorov-Smirnov test. Continuous data were expressed as mean and 95% confidence interval (CI) and categorical variables as absolute number and percentage. A univariate regression analysis was performed to verify the association between the BDI score and demographic, clinical, echocardiographic, and functional variables. A multivariate regression analysis was performed to determine the independent predictors of depressive symptoms; those variables with a significance level less than 10% (p<0.1) were included in the final model.

## RESULTS

Fifty-nine patients were recruited for the present study. Out of these, 14 patients were excluded because they had non-cardiac forms of Chagas disease, six were using antidepressant medications, two had cognitive impairment, one had significant mitral stenosis associated with Chagas disease, and one was not able to perform the exercise test due to advanced knee arthrosis. Thus, 35 patients with ChC fulfilled the inclusion criteria and were evaluated. The average score found after administration of the BDI was 9 (95% CI: 6-12). In the sample, 13 (37%) patients had depression. Among these, 62% had mild-to-moderate depression and 38% had moderate-to-severe depression. None of the participants presented with severe depression. The average left ventricular ejection fraction was 54 (95% CI: 48-59), which characterized preserved cardiac function. The clinical, demographic, echocardiographic, and functional characteristics of the patients are shown in Table 1.

**TABLE 1:** Characteristics of the participants included in the study (n = 35).

Variable	Mean (95% CI), n (%)
Age (years)	48 (44 - 51)
Male sex (n)	23 (66)
NYHA functional class	
I	20 (57)
II	9 (26)
III	6 (17)
Clinical classification	
Stage B1	20 (57)
Stage B2	15 (43)
BMI (kg/m^2^)	27 (25 - 28)
SBP (mmHg)	102 (98 - 107)
DBP (mmHg)	67 (64 - 69)
HR (bpm)	67 (63 - 70)
HAP (score)	84 (81 - 86)
SF-36 physical summary (score)	48 (45 - 52)
SF-36 mental summary (score)	48 (42 - 55)
Mini-Mental State Examination (score)	24 (22 - 25)
VO2peak (ml.kg.min)	26 (23 - 29)
VE/VCO2 slope	32 (30 - 33)
6MWT (m)	548 (518 - 578)
LVEF (%)	54 (48 - 59)
LVDd (mm)	55 (51 - 59)

Data are presented as mean and 95%. **CI:** confidence interval. **BMI:** body mass index; **SBP:** systolic blood pressure; **DBP:** diastolic blood pressure; **HR:** heart rate; **HAP:** Human Profile Activity; **VO2peak:** peak oxygen uptake; **VE/VCO2 slope:** minute ventilation/carbon dioxide production relationship; **6MWT:** six-minute walk test; **LVEF:** left ventricular ejection fraction; **LVDd:** left ventricular end-diastolic diameter.

When the sample was stratified, the group of patients with depression had a higher body mass index, lower physical activity level, and lower score in the mental component of the SF-36 when compared to the patients without depression. No differences were observed in the clinical, echocardiographic, and exercise tests. The differences between the groups are shown in Table 2.

**TABLE 2:** Demographic, clinical, echocardiographic, functional, and quality of life variables stratified by the categories of depressive symptoms.

Variables	With depressive	Without depressive	p-value
	symptoms (n = 13)	symptoms (n = 22)	
Age (years)	49 (44 - 54)	47 (43 - 51)	0.753
Male sex (n)	7 (54)	16 (73)	0.220
NYHA functional class			0.208
I	7 (54)	13 (59)	
II	2 (15)	7 (32)	
III	4 (31)	2 (9)	
BMI (kg/m^2^)	28 (26 - 31)	26 (23 - 28)	**0.042**
SBP (mmHg)	100 (94 - 106)	104 (97 - 111)	0.693
DBP (mmHg)	67 (63 - 72)	66 (63 - 70)	0.490
HR (bpm)	64 (58 - 70)	69 (64 - 73)	0.135
HAP (score)	79 (76 - 82)	86 (83 - 90)	**0.002**
SF-36 physical summary (score)	48 (42 - 55)	48 (44 - 53)	0.662
SF-36 mental summary (score)	31 (22 - 40)	58 (54 - 62)	**<0.001**
Mini-Mental State Examination (score)	23 (21 - 25)	24 (22 - 26)	0.184
VO2peak (ml.kg.min)	24 (18 - 30)	28 (24 - 31)	0.491
VE/VCO2 slope	34 (31 - 36)	31 (29 - 33)	0.390
6MWT (m)	533 (475 - 591)	557 (520 - 595)	0.689
LVEF (%)	51 (40 - 63)	55 (49 - 61)	0.608
LVDd (mm)	57 (48 - 66)	54 (49 - 59)	0.701

Values highlighted in bold were statistically significant (p-value <0.05).

In the univariate analysis, female sex, NYHA functional class, body mass index, HAP score, mental summary of SF-36, peak oxygen uptake, and 6MWT distance were associated with the BDI score. In the multivariate model, only the HAP score and the mental summary of SF-36 remained as independent predictors of the BDI score in patients with ChC. The adjusted r^2^ of the model, including the two variables, was 0.66. The results of the univariate and multivariate analyses are shown in Table 3.

**TABLE 3:** Univariate and multivariate predictors of BDI scores in patients with ChC.

Variables	Univariate analysis			Multivariate analysis			
	R	Beta-coefficient	95% CI	p-value	Beta-coefficient	95% CI	p-value
Constant	-	-	-	-	66.214	44.689 to 87.739	**<0.001**
Age	0.137	0.13	-0.23 to 0.50	0.431	-	-	-
Female sex	0.399	6.60	12.00 to 1.22	0.018	-2.222	-6.685 to 2.242	0.313
NYHA class	0.411	4.23	0.91 to 7.56	0.014	0.064	-4.535 to 4.663	0.977
BMI	0.440	0.79	0.21 to 1.37	0.009	0.177	-0.324 to 0.678	0.472
SBP	0.070	0.04	-0.18 to 0.27	0.690	-	-	-
DBP	0.223	0.25	-0.13 to 0.62	0.197	-	-	-
HR	0.208	-0.17	-0.47 to 0.12	0.231	-	-	-
HAP	0.631	-0.72	-1.06 to -0.39	<0.001	-0.533	-0.804 to -0.262	**<0.001**
SF-36 physical summary	0.074	-0.07	-0.41 to 0.27	0.682	-	-	-
SF-36 mental summary	0.694	-0.35	-0.48 to -0.22	<0.001	-0.269	-0.386 to -0.153	**<0.001**
Mini-Mental State Examination	0.283	-0.50	-1.11 to 0.11	0.105	-	-	-
VO2peak	0.313	-0.31	-0.65 to 0.03	0.071	0.177	-0.505 to 0.450	0.907
VE/VCO2 slope	0.217	-0.41	-0.24 to 1.06	0.211	-	-	-
6MWT	0.315	-0.03	-0.06 to 0.01	0.066	-0.016	-0.048 to 0.015	0.293
LVEF	0.125	-0.07	-0.26 to 0.12	0.481	-	-	-
LVDd	0.121	0.08	-0.16 to 0.33	0.489	-	-	-

Variables in bold in the univariate analysis (p-value <0.10) were included in the multivariate linear regression model.

## DISCUSSION

To the best of our knowledge, this is the first study to investigate the clinical, echocardiographic, functional, and quality of life factors associated with depressive symptoms in patients with ChC. Our results demonstrated that: (1) depressive symptoms were prevalent in the study population, with a rate of 37%, (2) physical activity level, assessed by the HAP score, and mental health, assessed by the mental summary of SF-36, were strongly related to depressive symptoms in patients with ChC. 

Depressed patients have decreased life expectancies, and cardiovascular disease may be one possible explanation for the increased risk of premature death in these patients^33^. In the context of Chagas disease, a qualitative study reported that the initial discussions among respondents were regarding their experiences or fears about the disease and the possibility of death^34^. The disease was characterized as a precursor to high depression levels, possibly because of the risk of sudden death related to the cardiac form^35^. In the present study, depressive symptoms were found in 37% of patients with ChC. Ozaki et al.^16^ verified that in a sample comprising patients with different clinical forms of the disease, the prevalence of depressive symptoms was approximately 41%. The findings of both the studies were similar and highlighted the high prevalence of such symptoms in this population.

We also found that female sex, body mass index, functional performance, physical status, and mental health were associated with depressive symptoms. Previous studies^36^ ^,^ ^37^ have already shown a higher prevalence of depressive symptoms in women with ChC when compared to men, which has also been observed in other populations^38^ ^,^ ^39^. Sex differences in biological, psychological, and environmental factors, as well as many hormonal changes caused by the menstrual cycle, pregnancy, postpartum period, abortion, and menopause may explain the association of the female sex with depressive symptoms^40^. The body mass index is closely linked to self-esteem. Functional status was strongly associated with depressive symptoms in cardiac patients, and it can be a valuable tool in identifying patients at risk of hospitalization^41^. However, in the final multivariate model, we found that only mental health and the physical activity level remained independent predictors of the BDI score.

The association between mental health and depressive symptoms can be explained by several factors such as fear of death, difficulty in accessing appropriate treatment, and mental stress emerging immediately after diagnosis^15^. In addition, Chagas disease is considered to be one of the most neglected tropical diseases. It is often associated with poverty and characterized by stigma, which in turn can be a psychological and social burden, leading to progressive social exclusion, reduced health-related quality of life, and mental disorders^42^. In a meta-analysis, Daré et al.^43^ demonstrated that the association between parasitic diseases (including Chagas disease) and mental disorders (anxiety, depression, bipolar disorder, and schizophrenia) were relatively high in developing and emerging countries. Thus, stigma must be considered an important component in the emergence of depressive symptoms, as it leads to despair, hopelessness, embarrassment, increased stress, and depression^44^. The concern about mental health in patients with ChC has grown in recent years. A recent longitudinal study^45^ showed that the mental component of SF-36 was an independent predictor of adverse cardiovascular events in these patients. This finding, together with the results of the present study, reinforces the need for clinical and psychological management of the mental health of patients with ChC.

The inverse association between physical activity and depressive symptoms is well established in the literature^46^. A population-based study with 26,615 middle-aged and older healthy participants at baseline investigated the associations of estimated cardiorespiratory fitness with depression and reported that a medium and high level of cardiorespiratory fitness was cross-sectionally and prospectively associated with lower odds of depression^47^. Moreover, physical inactivity has been shown to partially mediate the relationship between depression and mortality among persons with cardiovascular disease^48^ ^,^ ^49^. Results from the Cardiovascular Health Study, a multicenter prospective cohort study with community-dwelling older adults, showed that depressive symptoms and physical inactivity independently increased the risk of cardiovascular mortality and were strongly also associated with each other. In addition, individuals with both the conditions had a greater risk of cardiovascular mortality than those with only one condition^50^. In our study, patients with depression presented with a lower level of physical activity, and physical activity was found to be an independent predictor of depressive symptoms. Considering the effectiveness of exercise training in patients with ChC^51^ ^-^ ^53^ and taking into account the relationship between physical inactivity, depressive symptoms, and adverse health events related to cardiovascular disease, physical exercise programs should be considered for this population.

The present study had several strengths and limitations. As a limitation, our sample size was relatively small. In addition, our sample was composed predominantly of patients with preserved cardiac function, and the results should be limited to patients with this disease characteristic. As a strength, the present study demonstrated the association of physical activity and mental health with depressive symptoms in patients with ChC for the first time. 

In conclusion, depression is a prevalent condition in patients with ChC and predominantly preserved cardiac function. The physical activity level and mental health were found to be independent predictors of depressive symptoms in this population. Therefore, early interventions targeting these modifiable risk factors should be encouraged. 
